# A Collective Efficacy Intervention to Promote Community Resiliency: Protocol for a Quasi-Experimental Evaluation

**DOI:** 10.2196/93587

**Published:** 2026-07-21

**Authors:** Mary L Ohmer, Leah A Jacobs, Jason Beery, Anthony Fabio, Kim Falk, Ivana Gazarik, Christi Gomez, Tracey Joiner, Elizabeth Miller, Alex Neumann, Aaleah Oliver, Ross Reilly, Mohammad R Shedeed, April Zeiner, Alison J Culyba

**Affiliations:** 1 School of Social Work, University of Pittsburgh, 2204 Cathedral of Learning, 4200 Fifth Ave, Pittsburgh, PA, 15260, United States, 1 412-624-8214; 2 Nature Space Consulting, Pittsburgh, PA, United States; 3 Epidemiology Data Center, Department of Epidemiology, University of Pittsburgh, Pittsburgh, PA, United States; 4 Office of Behavioral Health, Allegheny County Department of Human Services, Pittsburgh, PA, United States; 5 Department of Pediatrics, School of Medicine, Division of Adolescent and Young Adult Medicine, University of Pittsburgh, Pittsburgh, PA, United States; 6 Division of Adolescent and Young Adult Medicine, Department of Pediatrics, University of Pittsburgh School of Medicine, Pittsburgh, PA, United States; 7 Neighborhood Resilience Project, Pittsburgh, PA, United States; 8 BlackteaBrownSuga Network, McKees Rocks, PA, United States; 9 University Center for Social and Urban Research, University of Pittsburgh, Pittsburgh, PA, United States

**Keywords:** community violence, youth violence, community-based participatory research, collective efficacy, community thriving

## Abstract

**Background:**

Community and youth violence are pervasive and have devastating health, economic, and social consequences. Violence too often impacts people of color and the life trajectories of youth in the United States, especially among people living in urban settings.

**Objective:**

In response, this study aims to evaluate the effectiveness of a novel *Community Resiliency Collective Efficacy Intervention* (*CRCEI*), which seeks to increase individual and neighborhood levels of collective efficacy, and reduce youth and community violence. The CRCEI has three phases: (1) community organizing and mobilization (engaging and working with diverse community members and collaborators to build capacity, plan, and recruit); (2) collective efficacy training (learning, discussing, and practicing relationship building, and facilitating thriving, resiliency, and strategies for organizing and intervening); and (3) community-based prevention projects (based on what participants learn in the training).

**Methods:**

The study was set in 8 urban, high-violence, racially segregated, and economically disadvantaged neighborhoods in Pittsburgh, Pennsylvania. Community partners and researchers selected 4 intervention sites based on perceived need and appropriateness. We then used participatory propensity score matching to select 4 comparison sites, which receive health education sessions in lieu of the CRCEI. The study assesses the impact of the CRCEI on participant-level collective efficacy and exposure to community violence (aim 1), community-level collective efficacy, and incidence of community violence (aim 2) via several data sources, including pre- and postsurveys with participants and community members, quantitative and qualitative data from observations and interviews, and secondary neighborhood-level data (eg, violence incidents and poverty rate). Data for aim 1 were collected from February 2023 to January 2025, and data for aim 2 were collected from September 2022 to May 2025. We complemented this impact analysis with an ethnographic process evaluation, which tracks fidelity and costs, describes implementation, and identifies facilitators and barriers across the intervention sites (aim 3).

**Results:**

We hypothesize that the CRCEI will provide a concrete, action-focused strategy to increase resiliency and reduce community and youth violence, with impact demonstrated by increased reports of collective efficacy and decreased exposure to violence at the individual (aim 1) and community levels (aim 2).

**Conclusions:**

Innovations of this project include testing a community-oriented and collective efficacy–oriented violence reduction intervention (vs criminal-legal and punishment-oriented strategies), implementation in participatorily identified and asset-mapped local settings (eg, neighborhoods for inclusion and community partners to host the intervention identified by community members as trustworthy), a capacity-building approach (eg, training facilitators from focal neighborhoods), and community member integration into collaborative team science. In this manuscript, we outline the rationale and design for the evaluation of this collective efficacy intervention.

## Introduction

### Background and Rationale

Youth and community violence are pervasive and negatively impact mental health and community well-being. In the Pittsburgh region, homicide rates increased by 43% in the city and by 27% in the county from 2019 to 2021, largely reversing the declining trends of previous years [1,2]. Violence disproportionately impacts Black communities, particularly young Black men. Despite making up only 6% of the county population, Black men are victims in 66% of annual homicides, and most are between the ages of 18 and 34 years. Overall rates of youth violence in Pittsburgh are similar to national-level data (eg, in 2023, 18% of 14‐ to 19-y-olds were in a physical fight in the past year, 11% carried a weapon in the past month, and 13% knew someone close to them who had been murdered) [2-4], and racial disparities in violence are profound, with homicide victimization rates 50 times higher among young Black men than the US average [3]. Exposure to violence has health, mental health, and social consequences, particularly in disadvantaged and minority communities [2]. Youth violence is associated with negative health and well-being outcomes across the life course, increasing the risk of behavioral and mental health difficulties, such as depression and suicide [4-8]. Youth violence can also negatively impact perceived and actual safety, participation in community events, and youth’s school attendance [4].

Youth violence is multifactorial in its sources. Among these sources, racism and economic inequality concentrate geographically and contribute to youth violence perpetration [9-13]. The deliberate concentration of Black individuals in neighborhoods and the subsequent divestment in those spaces have resulted in disproportionate exposure to psychosocial stressors (eg, discrimination and violence), with negative health outcomes across the life course, including mental health difficulties and substance use. Persistent experiences of bias-based discrimination (ie, racism, experiences of sexism, ableism, and homophobia) contribute to social isolation and emotion dysregulation, which, in turn, may contribute to substance use and mental health problems [5]. Ultimately, repeated exposure to early trauma and adversity such as violence and oppression can result in toxic stress responses that impede children’s healthy development. Simultaneously, structural racism contributes to constrained access to resources in communities, which impacts both youth and adults. Although the mechanisms for the association between racism and violence are not clear, discrimination may directly increase the likelihood of use of violence (ie, perpetration via isolation and emotion dysregulation) and indirectly through creating contexts of concentrated social disadvantage with a greater likelihood of exposure to violence.

Strength-based community-level strategies are essential to addressing structural racism, bias-based discrimination, and violence. A potential target of such strategies is *collective efficacy*. Collective efficacy is a neighborhood-level construct comprised of 2 interrelated components, including social cohesion (mutual trust) and informal social control, wherein neighbors support prosocial behavior and intervene in problems [14-16]. Research shows that engaging youth and adults to intervene in community problems fosters social connections, increasing residents’ ability to influence and control their environment and to promote community well-being [6-8]. Community organizing that directly addresses racial equity and structural changes to promote adolescent thriving is a promising strategy for building neighborhood resilience and protecting against violence [9]. Preliminary research found that a community organizing, collective efficacy-focused approach to prevent violence resulted in increased social cohesion among youth and adults, strengthened residents’ willingness to intervene to address violence, and improved attitudes around violence prevention [10]. However, evidence on the relationship between collective efficacy and violence primarily remains correlational. Addressing this gap, this study uses a quasi-experimental design to test the impact of a community-based program that aims to increase collective efficacy to reduce community and youth violence.

This quasi-experimental matched comparison group evaluation examines the effectiveness of the Community Resiliency Collective Efficacy Intervention (CRCEI). The primary objective is to test the effectiveness of CRCEI compared to health and wellness educational sessions on (1) participant-level collective efficacy and exposure to violence and (2) community-level collective efficacy and incidence of community violence. The study also aims to track the implementation of CRCEI across study sites, providing a detailed description of execution.

### CRCEI Intervention Description and Theoretical Frameworks

#### Community Resilience Collective Efficacy Intervention (Experimental Arm)

The CRCEI seeks to bring neighbors together, including youth and adults, build capacity to strengthen neighborhood collective efficacy, and ultimately reduce community violence. The 3-phase intervention brings youth and adults together to facilitate and strengthen trusting relationships, develop restorative and nonviolent intervention and organizing skills, and foster youth leadership and intergenerational partnerships. Consensus organizing strategies are used throughout the intervention to facilitate trusting relationships among neighbors and with community resources, thus building social cohesion, which is a key component of collective efficacy. Consensus organizing engages residents and external and internal resources to address neighborhood problems based on their mutual interests and concerns, which facilitates residents’ willingness to intervene [11,12]. A community-based approach offers opportunities to reach youth outside of school and to build natural mentoring relationships with adult participants and community facilitators.

**Figure 1.**
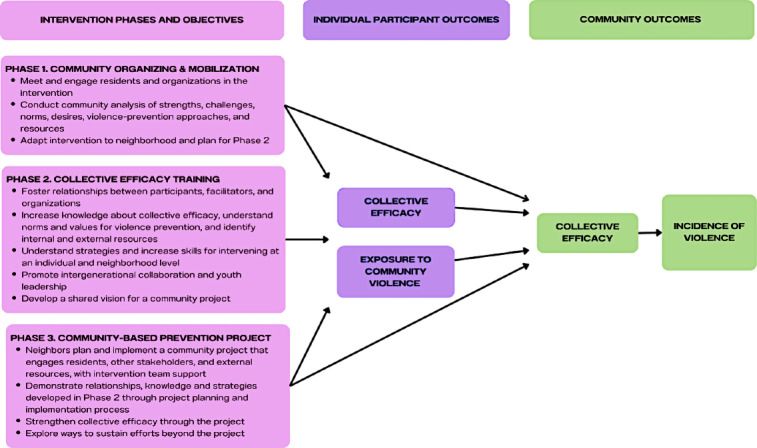
Overview of Community Resiliency Collective Efficacy Intervention intervention and outcomes.

As indicated in Figure 1, the intervention is implemented across 3 phases. Phase 1 includes conducting a community analysis to gather information on neighborhood history, assets or strengths and challenges, and current resources and efforts to address youth and community violence by meeting with and engaging community residents and organizations, attending community meetings and events, and gathering information on the neighborhood [10]. Residents’ perceptions of collective efficacy and violence exposure are assessed through a community survey. In addition, a community partner and resident facilitators are identified and engaged in this phase, including preparing for the phase II training by assessing facilitation skills, reviewing training content, and adapting it to unique community characteristics and issues, and recruiting residents for the training. Phase II is a 9-week training program that aims to foster collective efficacy among youth and adult residents, including identifying norms, values, and characteristics associated with youth and community violence, safety, and supportive connections in their neighborhoods. Results from the community survey are also shared and discussed. Participants learn and practice strategies for building relationships and safely intervening in neighborhood problems using restorative justice and nonviolent communication principles and neighborhood activism and organizing strategies for addressing structural causes of violence [13]. In phase III, the final phase of the intervention, a community-based prevention project aims to encourage youth and adults to apply newly developed skills to engage the broader community, bringing residents together to foster collective efficacy and reduce violence.

#### Theoretical and Empirical Basis for the CRCEI

The CRCEI draws on several theoretical frameworks: positive youth development, minority stress and ecosocial theories, and social disorganization theory. Positive youth development (PYD) posits (1) developmental plasticity (ie, young people can change and change their trajectory), (2) developmental assets exist both internally and externally (ie, the psychosocial conditions within which people live significantly influence well-being), and (3) developmental assets and a strength-based orientation are important for understanding and promoting development [14,15,17]. When assets are present and positive adaptation to life events is promoted, PYD holds that young people can thrive. Such thriving is marked by the “6 C’s of PYD,” or competence, confidence, character, connection, caring, and contribution (to self, relationships, and society more broadly).

Within the PYD framework, relationships matter. Adolescent-adult connections serve as important sources of social support that can foster racial-ethnic identity formation and can protect against violence involvement [8,18,19]. Particularly in the context of heightened police surveillance and systemic inequities, adult supports attuned to youths’ lived experiences can serve as “buffers and bridges” critical for envisioning life paths that diverge from violence [20]. Owing to PYD principles and this evidence, providing opportunities for meaningful and skill-building activities fosters adult and youth relationships, which create protective community environments that are a core component of youth violence prevention efforts and the CRCEI [21].

To address racial disproportionality in exposure to community and youth violence, the intervention draws on ecosocial theory and minority stress theory. Ecosocial theory underscores the extent to which structural racism contributes to constrained access to resources [22], while minority stress theory (MST) explains how racism can translate into disparities in health and well-being for individuals [5,23]. MST has been used to explain health disparities, positing that persistent experiences of bias-based discrimination (ie, racism, experiences of sexism, ableism, and homophobia) contribute to social isolation and emotional dysregulation, which, in turn, may contribute to substance use and mental health problems [5]. Chronic, repeated exposure to early trauma and adversity, such as violence and oppression, can result in toxic stress responses that impede children’s healthy development [24].

Findings from prior work in the study sites indicate that exposure to racism and bias-based discrimination is prevalent, with the majority of young people (82%) reporting at least one such instance (eg, “had the feeling that someone was afraid of you”; and “had people assume you’re not smart or intelligent” because of the color of your skin, language, accent, culture, or country of origin). Emerging evidence shows that interpersonal racism and discrimination prevention should include opportunities to promote racial or ethnic identity, increase awareness of unconscious bias (encouraging critical analytic thinking), shift social norms and policies to promote inclusive behaviors, and foster youth-adult connections [24,25]. Additionally, structural racism and discrimination prevention strategies should include adjustments to policies and practices that differentially impact specific groups of youth, as well as training in diversity and trauma-sensitive care. A critical need exists to test violence prevention interventions that directly bolster protective factors while addressing structural racism and discrimination at the community level.

Social disorganization theory posits that neighborhood-level characteristics (ie, the preponderance of social and economic disadvantage, residential instability, and family disruption) contribute to violence and other social and health problems, while collective efficacy (ie, the preponderance of social cohesion and social control) can protect against these problems [25,26]. Collective efficacy is a neighborhood-level construct comprised of social cohesion (mutual trust) and informal social control, wherein neighbors support prosocial behavior and intervene in problems [16,26]. Engaging youth and adults to intervene in community problems enhances social connections [27], increasing residents’ ability to influence and control their environment [28,29]. Intentional collaborations among youth and adults can increase collective efficacy and promote community well-being [28].

Theoretically, collective efficacy buffers the impact of structural inequality on violence and its sequelae. In neighborhoods where collective efficacy is high, unstructured socializing, antisocial behavior, delinquency, and violence tend to be low, as are violence-related mental health problems (eg, depression and anxiety), even after accounting for disadvantage and disorder [10,16,26,30]. Thus, interventions to increase collective efficacy have the potential to facilitate a variety of positive individual and community outcomes, but the links between collective efficacy and these outcomes are, to date, primarily correlational.

Together, PYD, ecosocial, and MST frameworks suggest that strengths-based, community-level strategies that incorporate young people and caring adults are essential for addressing structural racism, bias-based discrimination, and violence. Meanwhile, social disorganization theory and research indicate that collective efficacy is linked to lower levels of community violence and greater well-being. For these reasons, the CRCEI seeks to reduce community and youth violence by fostering adult-youth connections, raising awareness of structural factors and racism that influence community well-being, and building collective efficacy.

## Methods

### CRCEI (Experimental) and Health Sessions (Comparison) Arms

#### CRCEI (Experimental Arm)

As discussed earlier, the CRCEI takes place across 3 phases. In phase I (approximately 3 mo), *community mobilization and engagement* takes place. Researchers identify and partner with a community-based organization in each neighborhood whose mission, goals, and programs align with the intervention. The community partner and other community organizations help to identify 3 residents as community facilitators. This phase includes conducting a community analysis, a baseline community survey, and community engagement activities, attending community events and engaging with and getting feedback on the intervention from community residents and organizations. It also includes preparing the community partner and facilitators for the training, including reviewing and adapting training facilitation guides and case scenarios for role plays and other activities for each neighborhood, and recruiting youth and adult residents to attend the training.

Phase II is the *collective efficacy training*, which includes 9 training sessions over approximately 10 weeks (including a 1-wk break). The training location, dates, and times are discussed and decided with community partners and facilitators. The training sessions are held in accessible community locations, and dinner is provided at the beginning of each session. The youth present and get feedback on their community project ideas in the last session, and community resources are invited to share information and listen to the youth’s ideas. A community project planning committee is also formed in the last session.

In phase III (approximately 4 to 6 mo), the community partner and facilitators work with the community project planning committee to further develop and implement *a community project*. Each community receives a US $5000 grant to implement the project, which focuses on increasing collective efficacy and social connections, using intervening skills to take action on issues identified by participants in the training, providing alternatives to violence, particularly for young people, and increasing community resources. The project, selected by the community members, may include youth-centered community events and tangible products (eg, rehabilitation of recreational spaces) for young people and the wider community.

#### Health and Wellness Education Sessions (Comparison Arm)

The comparator for this intervention follows a similar cadence of interactions with community members, that is, an attention control. A combination of asset mapping and existing partnerships helps to identify a community organization in each comparison neighborhood to host psychoeducational sessions. With each neighborhood having its own unique character, shaped by history, demographics, and local culture, a list of health and wellness topics is developed based on the needs and wants of community members. This list of community needs and wants is generated through community surveying, discussions with the hosting community organization, and feedback from each wellness session. Topics for the health and wellness sessions include physical health practices and resources; mental health practices and resources; child and adolescent nutrition; public transportation resources; Alzheimer disease signs and treatment; and concussion prevention, signs, and treatment. A typical health and wellness session consists of a presentation as well as a question-and-answer session. Each session is led by a speaker from the university, health care delivery system, county, and community organizations (nonuniversity affiliated speakers receive an honorarium for participating), with expertise relevant to the topics identified. The location for the sessions is at a facility that is considered safe and trustworthy by community partners.

For this attention control design, both intervention and comparison sites involve approximately 18 hours of in-person time, generally spread out over 6- to 9-week periods. The program is delivered with some variation in schedules to meet the needs of community partners. Such configurations include, but are not limited, to, 2 health education sessions offered on the same day. We had different participation structures in the intervention and comparison sites to balance pragmatic concerns and resource constraints. The intervention was purposefully designed in prior pilot studies to be implemented with 3 structured phases in this study, therefore requiring more resources than the health sessions in the comparison sites.

#### Evaluation Design

The CRCEI was initially funded by the Substance Abuse and Mental Health Services Administration as part of a broader effort across the county to promote community mental health and increase resiliency. Subsequently, the team also received research funding from the Centers for Disease Control and Prevention to conduct a rigorous evaluation of this intervention (a cluster randomized controlled trial across similar neighborhoods is ongoing). As CRCEI was already being implemented in several neighborhoods to assess impact, a quasi-experimental matched comparison group design was used, using participatory propensity score matching to identify comparison neighborhoods with similar baseline characteristics as the sites already implementing CRCEI. Four neighborhoods receiving the CRCEI intervention are compared with 4 propensity-matched neighborhoods receiving the comparison health sessions. The study uses a community-based participatory and partnered approach. For this reason, we offered an alternative intervention in comparison communities rather than “care as usual” or “no intervention” to promote reciprocity. Furthermore, our aim was to match the demographics and characteristics of the communities and use the same data collection methods and measures in the intervention and comparison communities to analyze the aims for this study.

**Figure 2.**
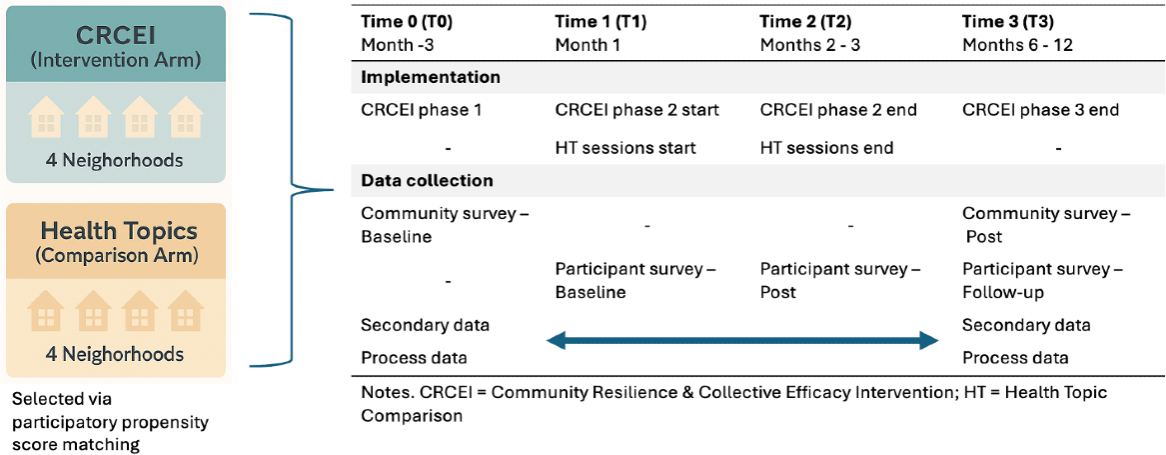
Study flow diagram. CRCEI: Community Resilience and Collective Efficacy Intervention; HT: Health Topic Comparison.

As illustrated in Figure 2, survey data were collected from participants in the intervention and comparison groups at 3 time points: baseline (T1; prior to exposure to the training or health sessions), post training or health sessions (T2; immediately following the 9-wk CRCEI training or last health session), and follow-up (T3; approximately 6 mo after the T2 to coincide with the completion of phase III in the intervention arm). To address secondary aims and inform neighborhood adaptation of the CRCEI, community survey data are collected at baseline (T1) and follow-up (T3), and secondary data on community and youth violence incidence are measured annually. In addition, an ethnographic process evaluation, including implementation science frameworks, is conducted to elucidate how CRCEI is implemented across intervention neighborhoods [31,32]. The evaluation focuses on dimensions of fidelity, reach, dosage, responsiveness, differentiation or characteristics, and adaptation and draws on a mix of observation, feedback questionnaires, and attendance, interview, and secondary data.

#### Site Eligibility Criteria, Selection Process, and Study Setting

This study includes 8 neighborhood sites in the greater Pittsburgh, Pennsylvania, region. Four neighborhoods were initially chosen as intervention sites based on having high levels of gun and community violence incidents and desiring or engaged in efforts to increase community safety and identified as priority neighborhoods by the Allegheny County Department of Human Services, Office of Behavioral Health, interested community partners, and the project Steering Committee. The Steering Committee guides and informs the project and includes community partners, resident facilitators, interdisciplinary and countywide partners, and research team members.

To identify 4 comparison sites, the team used a process we refer to as, “participatory propensity score matching.” Building on the work of the abovementioned stakeholder groups, the research team constructed a list of 29 additional neighborhoods that were similarly high in violence. Steering Committee members then rated the identified neighborhoods in terms of appropriateness and need, recommending the removal of 5 neighborhoods that were least comparable. The research team then calculated propensity scores for the remaining 24 neighborhoods.

The propensity scores were calculated based on measures used to construct the Social Vulnerability Index, which uses 15 social factors grouped into 4 related themes (socioeconomic status, household composition, race, ethnicity, or language, and housing or transportation) [33]. On the basis of local norms and steering committee input, we modified these slightly and included the following variables: population, percent below poverty level, percent age 65 years or older, percent age 17 years or younger, percent older than age 5 years with a disability, percent speaking English “less than well,” percent Hispanic, percent multiunit structures, percent mobile homes, percent crowding, percent on vehicle, and group quarters. Propensity scores were estimated for each neighborhood using a 15-covariate linear regression model, which included the census variables described earlier. Propensity scores were estimated using logistic regression in SPSS, and matching was performed with the FUZZY extension (version 2.0.1) using a nearest neighbor greedy algorithm with a caliper of 0.18. For each intervention neighborhood, the research team noted the top 4 comparison neighborhood matches. The research team then reviewed the recommended matching neighborhoods and data on those neighborhoods with the steering committee and identified the best match (based on score and steering committee knowledge of other factors) for each of the 4 neighborhoods already selected for intervention.

These 8 intervention and comparison neighborhoods struggle with poverty, school “push-out” (disciplinary actions that push youth, especially Black boys, out of the regular school system), and have among the highest rates of gun violence in the county (see Table 1 for neighborhood characteristics).

**Table 1.** Intervention and comparison neighborhood demographics. Absolute values are not available for all data in the table.

Neighborhoods	Tracts (n)	Population estimate	Minority, n (%)	Below 150% poverty (%)	No high school diploma (%)	No health insurance (%)	Aged ≥65 y (%)	Aged ≤17 y (%)	Single-parent households (%)
Intervention neighborhoods									
Westside city: Chartiers City, Crafton Heights, Elliott, Esplen, Fairywood, Sheraden, Westend	5	12,981	5979 (46.06)	37.56	9.68	5.98	13.04	22.30	10.40
Homewood (city)	4	7517	97.28	56.38	12.85	7.55	25.28	23.98	12.00
Braddock, North Braddock, Rankin	4	8536	67.93	39.38	9.10	6.55	18.28	22.05	14.50
Wilkinsburg	4	11,212	75.40	44.15	9.15	4.10	24.15	20.53	12.48
Comparison neighborhoods									
California Kirkbride, Manchester (city)	1	2864	75.40	29.10	4.20	4.90	12.30	15.80	10.90
East Hills (city)	1	2777	86.50	45.30	6.10	9.50	17.80	27.10	19.40
Turtle Creek	1	5161	40.00	38.80	9.60	4.40	23.30	22.40	17.30
Duquesne	3	5534	65.10	44.23	11.80	4.43	11.47	31.07	21.73

Within each neighborhood, a different organization works with the research team on the project. In the intervention neighborhoods, community partners whose mission, goals, and programs align with the project are engaged through a formal agreement and scope of work and receive funds to pay for identified staff to work with the research team on the project. In the comparison sites, a community organization is identified that can host and inform the health sessions and is given a stipend to assist with covering the time and effort for hosting the sessions at their facility. Community partners in the intervention neighborhoods include community development organizations, grassroots organizations working to promote safety and provide support for victims of violence and their families, or other neighborhood-based organizations working to improve community outcomes. In addition, 3 residents are hired as community facilitators in the intervention neighborhoods based on their engagement with youth and/or adult residents and in local safety and community organizing and improvement efforts. Community organizations hosting health sessions in the comparison communities include faith-based organizations, community centers, neighborhood associations, and libraries. Asset maps are created for each intervention and comparison neighborhood with the goal of identifying community champions and relevant resources to support the project. The research team relies on partnerships with key constituents in each of these sites (including site coordinators, facilitators, partners, and community members) to facilitate recruitment and retention as described below.

### Outcome Measures

#### Primary Outcome Measure

Table 2 contains a description of the primary and secondary outcome measures (Table 2). The primary outcome is self-reported collective efficacy at the individual participant level across 3 points in time (ie, T1, T2, and T3). *Collective efficacy* is measured with the 10-item Collective Efficacy Scale, which has been previously validated and widely used, including among low-income, African American, and youth samples [28,34,35]. The measure includes a 5-item “social cohesion and trust” subscale and a 5-item “informal social control” subscale. Each item is assigned values based on a 5-point Likert-style scale, and final scores are calculated by averaging responses across items (range 1‐5).

**Table 2.** Primary and secondary outcome measures.

Outcomes	Measures description	Time frame
Primary outcome		
Neighborhood collective efficacy (28, 34, 35)	Informal social control: How likely is it that your neighbors can be counted on to “do something” if.... Children were skipping school and hanging out on a street corner, Children were spray-painting graffiti on a local building. Children were showing disrespect to an adult, A fight broke out in front of their house, and someone was being beaten or threatened; The fire station closest to your house was going to be closed down by the city because of budget cuts, Youth are vandalizing or stealing a car in the neighborhood, and Someone is vandalizing your neighbor’s property. Five-point Likert scale from very likely to very unlikely. Social cohesion: Please indicate how strongly you agree or disagree with the following statements: People around here are willing to help their neighbors, This is a close-knit neighborhood, People in this neighborhood can be trusted, People in this neighborhood generally don’t get along with each other, and People in this neighborhood do not share the same values.” Five-point Likert scale from Strongly Agree to Strongly Disagree	Community survey T1 and T3; and participant survey T1, T2, and T3
Secondary outcomes		
Willingness to intervene (36)	How likely would you intervene in or “do something” about the following potential problems in your neighborhood? (Same items and Likert Scale as the Informal Social Control scale)	Participant survey T1, T2, and T3
Social connections (neighborhood integration) (10)	How many adults (age 18 and over) in your neighborhood… Do you know by sight? Do you know by name? Do you talk to on a regular basis? How many young people (less than age 18) in your neighborhood…Do you know by sight? Do you know by name? Do you talk to on a regular basis? Ordinal range (None (0), A few (1-5), Some (6-10), A lot (over 10))	Community survey T1 and T3; and participant survey T1, T2, and T3
Community norms, values, and attitudes about intervening (10)	How strongly do you agree or disagree with the following statements about your neighborhood? It is appropriate to question strangers in your neighborhood, It is appropriate to intervene in suspicious behaviors in your neighborhood, It’s OK to say something if someone is behaving inappropriately in your neighborhood, Good neighbors mind their own business, When neighbors intervene, neighborhoods are safer, I can identify behaviors that most of my neighbors would disapprove of, People in my neighborhood would support someone if they intervened in inappropriate behavior in the neighborhood, Personally assisting people in trouble is very important to me, The police play an important role in preventing crime in this neighborhood, It is the job of the police, not residents, to deal with inappropriate neighborhood behavior. Five-point Likert scale from strongly agree to strongly disagree.	Community survey T1 and T3; and participant survey T1, T2, and T3
Exposure to violence (37)	Composite measure comprised of Exposure to Violence and Witness to Violence to measure number of individual-level events of violence exposure. Response: Yes or No Exposure to violence: The next section asks about your personal experiences with violence in your neighborhood. Have you…been chased when you thought that you could really get hurt? been hit, slapped, punched, or beaten up? This does not include fooling around? been attacked with a weapon, like a knife or a bat? This does not include getting shot or shot at? been shot? This does not include being shot with any type of toy gun (ex. BB gun, paint gun, airsoft gun, pellet gun, etc.), been shot at, but not wounded? had someone threaten to seriously hurt you, other than what you have already shared? This includes being threatened with a weapon? seen a dead body? been told that someone you knew had been shot, but not killed? been told that someone you knew had been killed? Witness to violence: The next section asks whether you have seen someone else hurt by violence in your neighborhood. Have you… seen someone else get chased when you thought they could really get hurt? seen someone else get hit, slapped, punched or beaten up? This does not include when they were playing or fooling around, seen someone else get attacked with a weapon, like a knife or a bat? This does not include getting shot or shot at, seen someone else get shot? This does not include being shot with any type of toy gun (ex. BB gun, paint gun, airsoft gun, pellet gun, etc.), seen someone else get shot at, but not actually wounded? seen someone else get killed as a result of violence, like being shot, stabbed, or beaten to death? seen a gun in your neighborhood? heard gun shots in your neighborhood?	Community survey T1 and T3; and participant survey T1, T2, and T3
Neighborhood collective efficacy (26)	At the neighborhood level, we measure collective efficacy, using the same measure of collective efficacy described above. It is aggregated to a mean value based on responses among all participants within a given neighborhood on the community survey.	Community survey T1 and T3
Violent crime data	Pittsburgh Bureau of Police open data portal; Pennsylvania Uniform Crime Reporting (UCR) system and Allegheny County sources, including police blotter data and the county’s integrated data warehouse.	Community survey T1 and T3

#### Secondary Outcome Measures

The evaluation has 2 groups of secondary outcomes, those at the participant level and those at the neighborhood level. Intermediary, individual-level beliefs and behaviors related to collective efficacy are measured, including participants’ willingness to intervene, social connections, and prointervening norms and values. *Willingness to intervene* is a modified version of the Collective Efficacy social control subscale, with questions focusing on whether the participant is likely to intervene in social problems (ie, in lieu of whether they perceive their neighbors as being willing to intervene) [36]. *Social connections* are measured via 3 questions asking how many neighbors (youth and adults) that participants know by sight, name, and talking to on a regular basis (range 0 to >10). *Community norms, values and attitudes about intervening* is a scale asking participants the degree to which they agree with 10 statements related to norms and values pertaining to intervening in social problems [10]. *Exposure to violence* [37] is measured using 17 items with binary yes or no response options, including 9 items capturing *direct exposure* to violence and 8 items capturing *indirect exposure* to violence (ie, witnessing violence). This exposure to violence measure has been previously validated [37]. Exposure to violence values are calculated by the proportion of respondents endorsing each item (ie, responding “yes”). These data are collected from residents in the intervention and comparison neighborhoods via the community survey and at 2 time points, baseline and follow-up.

At the neighborhood level, we measure *collective efficacy* using the same measure of collective efficacy described earlier [26], which is aggregated to a mean value based on responses among all participants within a given neighborhood on the community survey. Prior research uses self-reported survey measures to measure collective efficacy at the individual and neighborhood levels. We measured collective efficacy at the individual level by surveying participants in the intervention and comparison sessions. We also measured collective efficacy at the neighborhood level through our community survey, and similar to Sampson et al, the responses are aggregated to the neighborhood level.

Finally, violent crime data for City of Pittsburgh neighborhoods are obtained from the Pittsburgh Bureau of Police open data portal, while data for municipalities outside the city (eg, Wilkinsburg, Duquesne, Turtle Creek, and Braddock) are drawn from the Pennsylvania Uniform Crime Reporting system and Allegheny County sources, including police blotter data and the county’s integrated data warehouse.

### Sample Size and Power

For the primary outcome in aim 1, individual-level neighborhood collective efficacy, the necessary sample size was calculated based on traditional methods that assume a fixed number of clusters. Using a 2-sided alpha of .05 and small-sample cluster-robust adjustments with 6 degrees of freedom, the study achieves 80% power to detect meaningful effects at the individual level. Specifically, the trial can detect a standardized mean difference of about 0.66 when the intraclass correlation coefficient (ICC) is 0.05. Under more conservative assumptions, the detectable effect size is approximately 0.83 when the ICC is 0.10 and 1.10 when the ICC is 0.20. These values highlight that the study is positioned to identify moderate to large individual-level intervention effects. By quantifying these detectable ranges, the trial not only clarifies its current analytic capacity but also establishes concrete guidance for future community trials, where additional neighborhoods or larger enrollments could be used to target smaller effects of substantive interest.

For aim 2, which evaluates neighborhood-level outcomes such as collective efficacy and community violence, the unit of comparison is the neighborhood. The study includes 8 neighborhoods: 4 assigned to the intervention and 4 to the comparison. With 8 clusters, the design is best suited to identifying larger neighborhood-level effects, which ensures that any observed changes will be both meaningful at the community scale and informative for the field. Using a 2-sided alpha of .05 and small-sample cluster-robust adjustments with 6 degrees of freedom, the minimum detectable effect size was estimated. Without adjustment for baseline, the detectable standardized effect is approximately 1.8. As aim 2 analyses will adjust for baseline neighborhood measures, precision improves as the correlation between baseline and follow-up increases. For plausible baseline–follow-up correlations, the detectable effect sizes range from approximately 1.9 when the correlation is 0.60, to 1.7 when the correlation is 0.70, to 1.4 when the correlation is 0.80, and to 1.2 when the correlation is 0.85. Reporting these estimates provides a transparent picture of what can be inferred from this trial and creates clear parameters for planning future evaluations, where additional neighborhoods would allow the detection of smaller, yet still important, community-level changes.

### Participant Eligibility Criteria

#### Community Survey Eligibility Criteria

Eligibility for participation in the community survey requires that participants reside in the specified neighborhood, meaning that they hold an address within the preselected community or communities, and speak English. Initially, only individuals aged ≥18 years were allowed to complete the survey, but this enrollment criterion was later amended, beginning in Homewood in November 2023, to include individuals aged ≥13 years due to the importance of understanding youth experiences and perspectives.

#### CRCEI and Health Session Participant Eligibility Criteria

Youth aged ≥13 years and adults who speak English and live within the identified neighborhoods are eligible to participate. Individuals can participate in the CRCEI or Health Sessions without participating in the research procedures.

Individual participants in the intervention training and the comparison health sessions are sampled by availability. Comparison and intervention sites use the network of community partners identified in the asset maps created for each neighborhood. This includes site leaders, program facilitators, recruiters with strong connections to their community, prevention specialists embedded in schools, and tabling at community outreach events. In the intervention sites, the research team also works collaboratively with community partners and facilitators, as well as community residents and organizations engaged in phase I of the intervention to recruit youth and adult residents to participate in the training. In addition, each community partner staff and resident community facilitator personally identifies and recruits residents in their personal and professional networks. Participant recruitment started in February 2023 and continued until the eighth neighborhood completed enrollment, through November 2024.

Individual youth and adult participants in the community survey are sampled randomly and by availability. In the first 2 intervention neighborhoods, availability sampling that primarily relied on facilitator recruitment did not yield adequate response rates and raised concerns about sampling bias. The team then adapted the data collection strategy and moved toward random sampling, adapting the Centers for Disease Control and Prevention’s CASPER model [38]. A dataset of residential addresses from county parcel, address, and property assessment data was assembled, and 30 residential addresses were randomly selected [39], their related US Census blocks were extracted, and those blocks were used to survey each of the 8 participating neighborhoods. The research team canvasses door to door, inviting participation in the community survey from each residence within each selected block. The number of blocks across the 8 neighborhoods ranges from 12 to 28 blocks. The number of addresses per neighborhood ranges from 524 to 2403. Given time limitations to the team’s ability to visit addresses multiple times, inability to reach some residents at home, and underrecruitment of community survey participants, random sampling is supplemented with recruitment of participants in public areas and community events (ie, availability sampling).

### Ethical Considerations

For the intervention and comparison groups, youth receive a description of the research study and a parental letter about the study. The parent letter includes an option for parents or caregivers to decline their child’s participation. The study was approved by the University of Pittsburgh Institutional Review Board (STUDY221001093). The Institutional Review Board approved a waiver of parental permission and a waiver of signed written consent. Research assistants (RAs) review the verbal consent form with participants at the beginning of the first intervention training and comparison health sessions and answer any questions pertaining to confidentiality, the program flow, and survey time points. The consent form covers all 3 waves of data collection, T1 through T3 as described earlier. For the community survey, verbal consent is obtained from all youth and adult participants.

### Retention in Program and Research

Once youth and adults are recruited, retention throughout the program is a key focus. Upon enrolling in either the comparison or experimental arm, prospective participants receive program information and complete a contact information sheet. RAs ensure that the document is legible and that all fields are completed. RAs use this information to contact the participant prior to each session to remind them of the session. Retention in the program is also promoted by compensating participants US $25 for attending each session. In the intervention sites, an additional US $25 is provided if participants attend all training sessions. New participants cannot enroll in the intervention training sessions after session 2.

Retention for participant survey data collection is facilitated by collecting detailed contact information and offering incentives for survey completion (US $25 for T1, US $25 for T2, and US $25 for T3; see Figure 2 for study flow). Participation in T2 and T3 surveys is facilitated via tracking participants with the help of community organizations that host the health sessions in the comparison sites and community partners and facilitators in the intervention sites. Participants provide detailed contact information at baseline to facilitate follow-up. Contact information is confirmed again at sessions following the baseline survey and at the T2 survey. Participants are also called or texted periodically by RAs between follow-up surveys to ensure that contact information is still valid. Finally, for those who miss a survey in the appropriate time frame, a comprehensive “make-up” survey is offered (with the same monetary compensation) to update contact information and increase the likelihood that they will participate in the next survey.

### Data Collection, Management, and Analysis

Three types of data are collected for this study, including survey data (participant and community), secondary data (on violence and community context), and implementation data (including observations, participation tracking, interviews, memos, and feedback surveys). As described below, these data are collected to capture outcomes at 2 levels of analysis and across multiple time points, as well as implementation across sites and time.

### Data Collection and Sourcing

For the purpose of measuring the individual-level outcomes (collective efficacy and exposure to violence) and covariates, survey data are collected across the following 3 time points: baseline (T1), the end of the phase II intervention training sessions, and comparison health sessions (T2), and follow-up approximately 8 to 12 months after baseline and to coincide with the completion of the community project for the intervention group (T3). The surveys are anonymous and linked by a personal study code that participants create by answering a series of questions that only they know the answer to at the beginning of each survey. This method of using a personal study code was selected to ensure anonymity and increase the likelihood of honest responses [40], especially for questions related to the use of violence. Baseline surveys are completed in person using paper surveys or tablets, depending on preference; T2 and T3 surveys are also completed in person on paper or a tablet or remotely using survey links that are texted or emailed to participants.

Secondary outcomes (neighborhood collective efficacy and incidence of violence) are measured at the neighborhood level. Neighborhood collective efficacy is captured through a community survey conducted at 2 points in time (T1 and T3). Incidence of violence is sourced from violent crime data for City of Pittsburgh neighborhoods obtained from the Pittsburgh Bureau of Police open data portal, while data for municipalities outside the city (eg, Wilkinsburg, Duquesne, Turtle Creek, and Braddock) are drawn from the Pennsylvania Uniform Crime Reporting system and Allegheny County sources, including police blotter data and the county’s integrated data warehouse. Incidents are geocoded, counted within neighborhood US Census tracts, and normalized by the population size for those tracts.

In addition to data collected for outcome analyses, data are collected to understand how the CRCEI is implemented, how implementation varies across neighborhoods, and factors affecting implementation, focusing on dimensions of fidelity, responsiveness (including engagement, satisfaction, and impact), reach, dosage, characteristics, and adaptation. Data sources include (1) feedback questionnaires completed by participants after the end of each CRCEI (note: participants in the health topics sessions also provide feedback, and those data are not part of the implementation science study); (2) semistructured observation forms completed by RAs during each CRCEI training session; (3) interviews with CRCEI team members (after every 3 neighborhoods) and community partners and facilitators (after phase II in each neighborhood) conducted by a project manager or coinvestigator; (4) confidential, semistructured interviews with CRCEI participants (between phase II and T3 data collection) conducted by a project manager or coinvestigator; (5) unstructured field notes from CRCEI team meetings collected by a coinvestigator; (6) attendance records documented by RAs during all training sessions; and (7) secondary data sources on community contexts (eg, US Census data and community violence data). Interviews with CRCEI team members, participants, and community partners and facilitators focus on experiences with the program and are used to guide ongoing implementation, including sustainability in the participating neighborhoods. Interviews with CRCEI team members also provide insight into adaptations, characteristics, and barriers to and facilitators of implementation. Qualitative data are complemented with quantitative data from the feedback questionnaires. Attendance records during each session speak to reach, dosage, and responsiveness (engagement). Finally, secondary data are sourced from the US Census and local police data to understand contextual features of each neighborhood.

### Data Management

Participant surveys and community surveys are primarily web-based (backup paper surveys are used as needed) on tablets using REDCap (Research Electronic Data Capture) hosted at the University of Pittsburgh, an online data management and survey system [41]. Responses to the anonymous web-based secure survey are entered by the participants themselves on an electronic tablet; no data are stored on the computers themselves. Only research staff who have been added to the project can access this online database. Data are downloaded and stored on a password-protected shared drive that can only be accessed by users with the appropriate permissions. No names are connected to the survey data, as each participant creates their own secret code as described earlier.

The only study documents that contain unique personal identifiers are contact forms and the contact list of participants to assist with recontacting participants for follow-up surveys. Contact forms are stored in a secure file drawer inside the locked office of the principal investigator (PI) whenever not in use. Contact forms are stored separately from any survey data collected in this study (the survey data are collected via computer and immediately housed in a password-protected secure database). The names of participants are kept in encrypted files on a password-protected server behind the university computer system’s firewall.

For implementation data, quantitative data from feedback surveys and observations are initially entered into REDCap and a programmatic database, also located on the password-protected shared drive. They are then exported and imported into Dedoose, a qualitative data analysis software (2025), where qualitative and quantitative indicators can be integrated by study site for the purposes of analysis.

### Analytic Methods

#### Statistical Analyses

Descriptive statistics will be used to summarize the sample with regard to baseline characteristics of interest. Means and SDs will be presented for continuous variables, while sample proportions will be provided for categorical variables. Ninety-five percent CIs will accompany all sample statistics.

#### Analytic Approach

Analyses are tailored to the quasi-experimental design (with considerations for clustered data by neighborhood) and aligned with the study aims. The primary independent variable of interest is treatment assignment at the neighborhood level (intervention vs comparison). Dependent variables include both individual-level outcomes (eg, collective efficacy and exposure to community violence) and neighborhood-level outcomes (eg, collective efficacy aggregated to the neighborhood, police-reported crime, and violence rates). Models will incorporate both fixed effects (eg, treatment, baseline values, and covariates) and random intercepts to account for clustering where appropriate. Inferences will rely on cluster-robust SEs with small-sample corrections (df=6). Effect sizes and 95% CIs will be reported throughout, with emphasis on estimation and interpretation of magnitude rather than binary hypothesis testing.

#### Aim 1 (Individual-Level Outcomes)

The individual is the unit of analysis, but clustering within neighborhoods is explicitly modeled. The independent variable of interest is neighborhood-level treatment assignment, with covariates including baseline individual scores, demographic characteristics (eg, age, sex, race or ethnicity, and education), and relevant neighborhood-level indicators. Continuous dependent variables will be analyzed using linear mixed effects models of the form:


Yij=β0+β1(treatmentj)+β2(baselineYij)+β3Xij+uj+εij,

where YijY_{ij}Yij is the outcome for individual iii in neighborhood jjj, XijX_{ij}Xij are individual covariates, uju_juj is a random intercept for neighborhood, and εij\varepsilon_{ij}εij is the individual-level error term. The random intercept uju_juj captures unobserved contextual influences shared by individuals in the same neighborhood (eg, social environment, policing, or community resources). By including uju_juj, the model allows each neighborhood to have its own baseline level of the outcome, and treatment effects are estimated net of this shared clustering. For binary dependent variables (eg, presence or absence of exposure to violence), generalized linear mixed models with a logit link will be used, also including a neighborhood random intercept.

The intercept term represents the expected outcome value when predictors are set to 0 (or to reference categories). In the context of these analyses, a random intercept for neighborhood allows the model to adjust for systematic differences between neighborhoods not explained by observed covariates. This ensures that individuals are not treated as statistically independent when they belong to the same neighborhood and that SEs correctly reflect the clustered design. Fixed intercepts anchor the regression line, providing the baseline level against which covariate and treatment effects are interpreted.

#### Aim 2 (Neighborhood-Level Outcomes)

The neighborhood is the unit of analysis. The dependent variables are neighborhood-aggregated outcomes (eg, mean collective efficacy scores and rates of crime or violence). The independent variable is treatment assignment. The primary analysis will use an analysis of covariance specification, where the neighborhood-level outcome at follow-up is regressed on treatment assignment and the baseline value of the same outcome. This model provides an adjusted mean difference in follow-up outcomes between intervention and comparison neighborhoods, controlling for baseline heterogeneity. As only 8 clusters are available, models will not include random effects but will instead rely on cluster-robust variance estimators with small-sample corrections. Adjustment for additional neighborhood-level covariates (eg, baseline socioeconomic disadvantage, racial composition, and baseline crime levels) will be included cautiously in sensitivity analyses, given the limited degrees of freedom. These covariates will be added one at a time to assess the robustness of the primary results. Sensitivity analyses will also include difference-in-differences models, where the dependent variable is the change from baseline to follow-up. Results will be expressed as standardized effect sizes for comparability across outcomes and to provide planning parameters for future trials.

#### Missing Data

Missing data will be addressed using full information maximum likelihood in the mixed effects models, which provides unbiased estimates under a missing-at-random assumption. As a sensitivity analysis, multiple imputation by chained equations will also be conducted for key outcomes and covariates. Both approaches preserve variability and prevent the downward bias in SEs that can arise with complete-case analysis. The extent and patterns of missing data will be reported, and the results will be compared across approaches to confirm robustness.

### Process Evaluation Analytic Methods

A mixed methods convergent approach is taken to analyze process data across quantitative and qualitative sources [42] (see Table 3 for a full description of implementation dimensions and data). This approach involves concurrent analysis of quantitative and qualitative data. Specifically, basic descriptive statistics are used to quantitatively describe implementation across intervention sites and to indicate fidelity or lack of fidelity to core program components. Simultaneously, qualitative data are integrated across sources, analyzed using Willig et al’s 6-step approach to thematic analysis [42-47] and integrated into quantitative results on an ongoing basis. Dedoose is used to organize, code, memo, and visualize qualitative and quantitative process data. Bringing both quantitative and qualitative findings together, implementation indicators are verified across data sources and quantitative findings are explained in more depth by findings from qualitative analyses. Visual tools, including joint displays of qualitative and quantitative data, are used as an analytic aid [45], especially in identifying patterns and unique cases. All findings related to implementation indicators are tracked in an implementation matrix that was collaboratively designed by the second author and intervention team [47]. In alignment with principles of data justice and guidance on enhancing trustworthiness in qualitative research [13,37], findings are verified on an ongoing basis and via discussion with research team members, intervention and comparison team members, and participants (in interviews and steering committee meetings).

**Table 3.** Process and implementation outcomes for the intervention sites.

Implementation construct	Dimensions	Definition	Data sources	Timing
Fidelity: whether and how the intervention is implemented as intended	Phase 1 Fidelity	Whether and how phase 1 is implemented as intended	Session observation forms and memos	Each session in phase 2
	Phase 2 Fidelity	Whether and how phase 2 is implemented as intended	Team event calendar	Ongoing
	Phase 3 Fidelity	Whether and how phase 3 is implemented as intended	Facilitator interviews	Between phases 2 and 3
	Global fidelity	Whether and how the intervention is implemented as intended overall	CE^a^ team interviews Fidelity matrix Semistructured field notes and memos	Implementation midpoint and end Ongoing All programmatic meetings
Adaptations: modifications made to the intervention as designed to better fit the setting’s needs, preferences, or other important characteristics, as well as motivations; also speaks to differentiation across sites	Phase 1 Adaptations	Planned and unplanned modifications to the intervention in phase 1	Session observation forms and memos	Each session in phase 2
	Phase 2 Adaptations	Planned and unplanned modifications to the intervention in phase 2	Facilitator interviews	Between phases 2 and 3
	Phase 3 Adaptations	Planned and unplanned modifications to the intervention in phase 3	CE team interviews	Implementation midpoint and end
	General Adaptations	Planned and unplanned modifications to the intervention that span phases	Semistructured field notes and memos	All programmatic meetings
Responsiveness: the degree to which and ways participants engage/participate in, are satisfied with, and demonstrate or express uptake of content	Engagement (phase 2)	The degree to which and ways in which session content and delivery involved participants during and across sessions, includes indication of lack of engagement during sessions or missing sessions, and sentiments of inclusion or exclusion expressed by participants	Session observation forms and memos Feedback questionnaires Participant and facilitator interviews	Sessions in phase 2 Sessions in phase 2 Between phases 2 and 3
	Satisfaction (phase 2)	The degree to which participants and facilitators are satisfied with sessions	Feedback questionnaires	Each session in phase 2
	Sources of satisfaction	The elements of the program participants and facilitators enjoyed or otherwise met or exceeded expectations	Participant and facilitator interviews	Between phases 2 and 3
	Uptake of intervention content	Perceived or observed change among participants, facilitators, and the community in relation to intervention	Session observation forms and memos Participant and facilitator interviews	Each session in phase 2 Between phases 2 and 3
Reach: the proportion of participants that meet inclusion criteria, the representativeness of participants to the target population (e,eg, end users of the intervention) that accepts and uses the intervention (Durlak 2010), and the match between who participates; also includes perceptions of the ideal target population	Acceptance	The number of facilitators and participants recruited and proportion youth versus adults	Participant survey Facilitator contracts	T1 Phase 1
	Participant relevance	The proportion of participants and facilitators that meet inclusion criteria and reflect the broader neighborhood population Perceived facilitator and participant relevance to the target population	Participant survey Facilitator contracts CE team interviews US Census 2020 Participant and facilitator interviews CE team interviews	T1 Phase 1 Implementation midpoint and end NA Between phases 2 and 3 Implementation midpoint and end
	Meeting attendance	Participation of facilitators and participants in phase 1 (facilitators only), phase 2 (facilitators only), and phase 3 team and planning meetings, for youth and adults	Payment records Team event calendar	Ongoing Ongoing
	Session attendance	The number of sessions attended per participant and facilitator, youth, and adults	Attendance records Payment records CE team interviews	Phase 2 Ongoing Implementation midpoint and end
		Barriers to and facilitators of attendance	Participant and facilitator interviews CE team interviews	Between phases 2 and 3 Implementation midpoint and end
	Community reach	The number of households that participated in the community survey	Community surveys Community surveys	Phase 1 start and phase 3 end Phase 1 start and phase 3 end
		The number of invited speakers and service providing and other organization representatives that attended phase 2	Session observation forms and memos	Phase 2
		The number of community members who participated in or were touched by the phase 3 project and how	Attendance records	Phase 3
Dosage: how much intervention was delivered	Phase 1 Dosage	The number of community engagement activities conducted or events attended The number of phase 1 meetings held with facilitators and participants (phase 3 only) The number of community engagement activities (phase 1) conducted or events attended by a team member The number of households invited to participate in the community survey in phase 1	Team event calendar Team event calendar and payment records Team event calendar Community survey tracking forms	Phase 1 Phase 1 Phase 1 Phase 1 start and Phase 3 end
	Phase 2 Dosage	The number of training sessions held The number of phase 2 meetings held with facilitators	Session observation forms and memos Team event calendar and payment records	Phase 2 Phase 2
	Phase 3 Dosage	The number of planning meetings held with community members and facilitators The number of hours phase 3 project was implemented for and by community members	Team event calendar and payment records Team event calendar and payment records	Phase 3 Phase 3
Qualities and differentiation: features of implementation and the context that make each site unique	Neighborhood context	Social and economic conditions in the neighborhood	US Census Police violent crime incidence data Community survey memos CE team interviews Participant and facilitator interviews	2020 Monthly Phase 1, phase 3 Implementation midpoint and end Between phases 2 and 3
	Organizational context	Focus, size, structure, and other characteristics of partner organization	Semistructured field notes and memos CE team interviews Participant and facilitator interviews	Ongoing Implementation midpoint and end Between phases 2 and 3
	Significant events w/in sessions and group dynamics	Significant moments of conflict, celebration, and learning	Session observation forms and memos CE team interviews Participant and facilitator interviews	Phase 2 Implementation midpoint and end Between phases 2 and 3
	Significant events in the community	Significant crime, violence, or other events occurring in the community	Semistructured field notes and memos Session observation forms and memos CE team interviews Participant and facilitator interviews	Ongoing Phase 2 Implementation midpoint and end Between phases 2 and 3
	Competing interventions	Discussion or observation of other community-building and violence prevention interventions in the community	Session observation forms and memos Semistructured field notes and memos CE team interviews Participant and facilitator interviews	Phase 2 Ongoing Implementation midpoint and end Between phases 2 and 3

a CE: collective efficacy.

### Monitoring

Given the sensitivity of the questions being asked regarding violence perpetration, a Certificate of Confidentiality was received from the Centers for Disease Control and Prevention to protect the research data from subpoena. Extra precautionary measures are taken to protect the data, including the use of a personally created ID code to maintain anonymity of the survey data and an internal data safety and monitoring plan, which includes the following: (1) systematically review assessment materials to ensure that assessment is conducted appropriately and that participants disclosing abuse or violence during the course of taking the survey receive appropriate connection to violence-related services and that mandated reports are made by site personnel when appropriate; (2) systematically review notes from RAs to ensure that participants experiencing distress are being connected directly with the site directors and youth workers, receiving educational materials, and being referred appropriately; this includes ensuring that all RAs document asking each participant about emotional distress after completion of the survey; (3) monitor staff performance with regard to protection of privacy, confidentiality, maintenance of secure databases, and study procedures designed to reduce the risk of distress and potential breaches of confidentiality; (4) ensure that one of the co-PIs, or a designated qualified individual, is available by pager in case research staff need to confer regarding participants’ behaviors or comments made during a survey or other research activities; (5) ensure that one of the co-PIs, or a designated qualified individual, is available by pager in case educators or violence prevention advocates need to confer regarding participants’ or workers’ behaviors or comments made during study implementation (ie, during training, survey administration, or follow-up contact with site administrators, youth workers, and facilitators), and (6) review and report any adverse events associated with the study.

## Results

Four initial neighborhoods were already participating in CRCEI prior to receiving the grant to conduct a rigorous evaluation, as described earlier. Community organizations within the 4 neighborhoods selected for comparison agreed to participate in the study. Neighborhood characteristics are similar between intervention and comparison neighborhoods (Table 1). A total of 262 participants enrolled in the study neighborhoods (n=111, 42%, in the intervention [CRCEI], with an age range of 21‐36 y per site; n=151, 58%, in the comparison health sessions, with an age range of 25‐55 y per site); all enrolled in the study and completed a baseline survey (100% participation; n=262; k=8). Most intervention and health session participants identify as Black or African American (n=170, 65%) and were born in the United States (n=227, 87%). Nearly two-thirds identify as cisgender women (n=165, 63%), and ages range from 11 to 86 years, with a median age of 39 years. Nearly half have some education beyond a high school diploma (n=123, 47%) and were living without a partner (n=123, 47%). The primary and secondary outcomes will be analyzed using the outcome measures described earlier in the methods section. A mixed methods convergent approach will be used to analyze process and implementation outcomes across quantitative and qualitative sources.

The study was funded by SAMHSA for a 5-year period starting on October 1, 2021, and by the CDC for a 4-year period starting on October 1, 2022. Data collection for aim 1 of the study was conducted through intervention and comparison participant surveys beginning on February 27, 2023, and ending on January 15, 2025. Data collection for aim 2 was conducted through intervention and comparison community surveys beginning on September 9, 2022, and ending on May 15, 2025. Data analysis for aims 1 and 2 began in April 2026, and we expect to publish the results for aims 1 and 2 in 2027. Data analysis for aim 3 began in February 2023 and ended in May 2025. Analysis of the cumulative results from the implementation science study began in April 2026, and we expect to publish the results from the implementation science study in 2027.

## Discussion

This study is a community-based quasi-experimental evaluation of a CRCEI implemented in lower resource Pittsburgh neighborhoods that involves individual- and community-level outcomes. The comparison intervention offers health and wellness educational sessions on topics of interest to community members. The primary outcomes of the intervention are collective efficacy and exposure to community violence at the participant level. The secondary outcomes include willingness to intervene, social connectedness, and norms, values, and attitudes about intervening at the participant level and neighborhood collective efficacy and rates of community violence at the neighborhood level.

The strengths of this study are the rigorous approach combined with strong partnerships with multiple community partners, including community leaders, community agencies, religious institutions, libraries, school districts, and neighborhood associations, who facilitate recruitment and retention. Additionally, close attention to CRCEI intervention implementation will allow for exploratory analyses on factors that impact implementation and outcomes: organizational- and facilitator-level characteristics that contribute to high fidelity to the intervention; strategies community facilitators use for introducing and facilitating discussions; and barriers to and facilitators of implementation with fidelity.

The study also has limitations related to participant retention, measurement, and sampling. Two factors limit retention and, relatedly, data missingness. First, as a community-based study recruiting participants with multiple structural barriers to participation, including transportation, retention of this cohort remains a critical challenge, with the most vulnerable at especially high risk for loss to follow-up. Thus, there will likely be significant nonrandom missing data. The study team makes every effort to reach out to participants and reduce barriers to participation (eg, delivering paper surveys to their home). It is important to note that participants in the intervention training sessions are asked to attend all the sessions and are given an additional US $25 if they meet this goal. This additional payment helps with retention in the training sessions. However, additional participants cannot join after the second session. In comparison sites, participants can attend any or all the health sessions and can start attending any session, resulting in more total participants in the health sessions.

Participant surveys are collected anonymously, with each participant creating their own personal identification code that only they know to match surveys over time. Such user-identified codes are effective at deidentification and therefore reduce bias, such as social desirability responding. However, they also limit data analysis to those participants for whom records can be matched. We attempt to address this problem using a detailed matching algorithm across sites and time points [40]. We will conduct several sensitivity analyses to assess impact on results (see *Methods*). Second, 2 issues may affect data quality. Different modes of delivery (researcher and self) and formats (oral, paper, and online) are used for participant and community surveys. These differences in administration and instrumentation can threaten measurement validity by introducing unintended sources of response variation. In addition, secondary outcome measures (willingness to intervene, social connectedness, and norms, values, and attitudes about intervening) have not been formally validated. However, prior studies suggest criterion validity for these measures, finding intervention-related gains pre- and post-participation [10,48]. We will analyze the association of these measures with related and unrelated measures and conduct subgroup analyses with data from this study to strengthen confidence in measurement validity. Finally, the study cannot overcome limitations related to availability sampling of participants, as is common in similar interventional studies. However, the study takes an important step toward enhancing causal validity (via propensity score matching of neighborhoods), and thus, threats to generalizability engendered via availability sampling should minimally be accompanied by selection bias.

On the basis of neighborhood uptake and participant recruitment, intervention training sessions and health topic sessions are acceptable at a neighborhood and individual level and recruitment is feasible. The strong partnerships previously established with key stakeholders in each site facilitate neighborhood uptake and participant recruitment. Furthermore, participant and community surveys can be feasibly administered. Neighborhoods selected for the study reflect the intended neighborhoods and residents, that is, predominately BIPOC (Black, Indigenous and People of Color), low socioeconomic status facing multiple challenges, including violence.

In summary, this study protocol outlines the initial quasi-experimental evaluation of an ongoing federally funded collective efficacy intervention. Findings may provide urgently needed information about the effectiveness of a strengths-based program that focuses on resiliency building and collective efficacy for communities impacted by violence that is an alternative to deficit-based, punitive, and carceral approaches. The intervention and its results will also inform community interventions and policies that promote safe, supported, and connected communities and build their capacity for sustainable community change. Lessons learned with this evaluation also guide the implementation of an ongoing cluster-randomized controlled trial of the novel CRCEI in 12 additional communities in the Pittsburgh region.

## Supplementary material

10.2196/93587 Peer Review Report 1 Peer review report from ZCE1 MLW (13)—National Center for Injury Prevention Special Emphasis Panel CE22-013: Rigorous Evaluation of Community-Centered Approaches for the Prevention of Community Violence; National Center for Injury Prevention and Control (National Institutes of Health, USA).

## Abbreviations

BIPOC: Black, Indigenous and People of Color
CRCEI: Community Resiliency Collective Efficacy Intervention
ICC: intraclass correlation coefficient
MST: minority stress theory
PI: principal investigator
PYD: positive youth development
RA: research assistant
