# Supplementary material for: A Collective Efficacy Intervention to Promote Community Resiliency: Protocol for a Quasi-Experimental Evaluation
Source: JMIR Res Protoc. 2026 Jul 21;15:e93587. doi: 10.2196/93587 (PMC13387632; doi:10.2196/93587)
Supplement: Peer Review Report 1 [file resprot-v15-e93587-s001.pdf]

SUMMARY STATEMENT

(Privileged Communication)

PROGRAM CONTACT:
Amanda Garcia-Williams
(770) 488-3936
gvl8@cdc.gov

Release Date: **09/14/2022**
Revised Date:

Application Number: **1 U01 CE003524-01**

Principal Investigator

MILLER, ELIZABETH

Applicant Organization: UNIVERSITY OF PITTSBURGH AT PITTSBURGH

Review Group: **ZCE1 MLW (13)**
National Center for Injury Prevention Special Emphasis Panel
CE22-013: Rigorous Evaluation of Community-Centered Approaches for the Prevention of Community Violence

Meeting Date: **06/28/2022**
RFA/PA: **CE22-013**
Council: **AUG 2022**
Requested Start: **09/30/2022**

Project Title: **A community-centered collective efficacy intervention for prevention of community violence**
SRG Action: **Impact Score:38**

Human Subjects: **30-Human subjects involved - Certified, no SRG concerns**
Animal Subjects: **10-No live vertebrate animals involved for competing appl.**
Gender: **1A-Both genders, scientifically acceptable**
Minority: **1A-Minorities and non-minorities, scientifically acceptable**
Age: **1A-Children, Adults, Older Adults, scientifically acceptable**
NIH Defined Phase III Clinical trial

Project Year	Direct Costs Requested	Estimated Total Cost
1	425,893	553,454
2	421,121	547,253
3	421,206	547,364
4	420,647	546,637
TOTAL	1,688,867	2,194,708

1U01CE003524-01 Miller, Elizabeth

RESUME AND SUMMARY OF DISCUSSION: This application, by a seasoned Principal Investigator (PI), proposes to conduct a cluster randomized controlled trial (CRT) to evaluate the effectiveness of a novel community-centered intervention that promotes thriving and resiliency to reduce community violence. The specific aims are to: 1) use a CRT (20 clusters, with “neighborhood” as the unit of randomization; 30 participants/neighborhood) to assess the effectiveness of Community-Centered Approaches for the Prevention of Community Violence (CRCEI) on collective efficacy and community violence compared to controls; 2) examine the impact of CRCEI on participants; and 3) elucidate implementation facilitators and barriers from individual to neighborhood-level, including tracking fidelity to the intervention and costs associated with program implementation. Reviewers determined this proposal to have high potential for public health impact.

Major strengths of the application were identified by reviewers. In terms of significance, this is a very large-scale project in Pittsburgh that intends to investigate the development of community self-efficacy as a variable that is hypothesized to reduce youth violence (YV) engagement. The study would be implemented in segregated neighborhoods that are plagued by concentrated disadvantage. The proposed evaluation project is embedded within a larger effort known as The Pittsburgh Study (TPS) that is led by Dr. Miller and that already is funded by the Substance Abuse and Mental Health Services Administration (SAMHSA) and the National Institute of Mental Health (NIMH), which is a longitudinal study of biological, social, and environmental influences on child health and thriving. The specific intervention in the proposed study involves six guided discussions in each of the communities about child thriving, with a sample size of 30 participants per community. The intent is to discuss child thriving with the adults, followed by an invitation for them to participate in a six-month long collective efficacy intervention that is intended to promote neighborhood-level transformation through a community-based project to develop collective efficacy. The theory is that community self-efficacy will lead to reduced violence engagement in the communities. The sample size for the collective efficacy study is 20 neighborhoods with 30 participants per neighborhood overall, and control communities that will be offered health care education instead of the collective efficacy and child thriving intervention. Crime rates in the intervention communities will be compared with those in the control communities. The participatory elements of the project are significant and suggest that the community intervention might be meaningful to community members. This application specifically addresses racism and structural discrimination as they relate to violence, which is important and compelling. The focus on community assets and strengths, as well as the use of asset mapping to determine safe settings for the intervention, is laudable. Use of community members as facilitators is an appealing aspect of the proposed project as well. The proposed study draws from Fabio’s research on community-level variables that impact levels of violence. This is an important level to examine, especially for this Notice of Funding Opportunity (NOFO). Fabio’s research found that racial segregation contributes more than individual risk factors to community violence, which suggests that the proposed study could be innovative. The investigators are highly qualified social scientists and pediatricians who are in an environment that is highly supportive of innovative and rigorous research. The PI is very strong and experienced in directing grants. The team is multidisciplinary, including a pediatrician who specializes in adolescent medicine, and MD/MHP who is the PI of the TPS, social workers specializing in community organization, and biostatisticians. The team has established in-depth community partnerships and collaborators to build strengths in different areas. The medical center already has a Youth Research Advisory Board that meets monthly to provide input on clinical and community-based research activities relevant to adolescent and youth health. Including youth in this way and specifically developing youth leadership skills is a valuable contribution. The strong involvement of community members and the Youth Research Advisory Board, who are compensated, speaks to how the team is striving to understand community members’ struggles and needs for support, as well as trying to make the research relevant to their values. This team seems well-situated to respond to this type of NOFO, which values community engagement, and makes a good case that the proposed research specifically aligns

with CDC priorities. A highly innovative aspect of the proposed work is that the team has tapped into some of the existing neighborhood institutions, including some neglected resources in violence prevention, such as libraries and faith-based institutions. It seems like they are trying to expand the notion of pre-mobilizing the community beyond police and teachers. In terms of the approach, the CRT design is rigorous. The dependent variables are primarily community collective self-efficacy and publicly reported incidents of crime in each community using multiple measures. The scientific environment is exceptionally strong, extremely well-resourced, and has been up and running for a while.

Weaknesses also were identified by reviewers. Regarding significance, it is not clear whether the community members would see this as helpful to preventing youth violence. There does not seem to be data from the community to suggest that community members would regard collective self-efficacy as a mechanism for preventing youth violence. The evidence cited from the literature does not address that either. Papers by Ohmer and the other Co-PIs posit that they can build community self-efficacy, but they do not actually demonstrate that community self-efficacy prevents violence with any empirical data or theory. Studies by Papachristos and Anderson indicate that most community members in high-crime, high-poverty communities of color are not violence-engaged. The people most likely to participate in this are not the high-risk group of those engaged in violence. It is not clear that the community members themselves would theorize that this would work to prevent violence, because there is more evidence in participatory studies that community members attribute violence to larger structural variables, such as mass incarceration and poverty, and that the collective self-efficacy model does not fix those problems at all. In terms of the approach, the conceptual model and hypotheses are lacking in their theoretical and empirical bases for focusing on community efficacy as a means of preventing violence. Insufficient attention is paid to attrition. Given that neighborhoods can be highly mobile, the sample size could be negatively impacted if attrition reaches approximately 50%, which it often does. However, this concern is tempered by the fact that the team will train facilitators from the focus neighborhoods to build capacity and sustainability, which might reduce some of the concerns about attrition. Further clarification of the research questions, methods, possible outcomes, and interpersonal processes and how they might interact would have been beneficial in terms of how, why, and under what conditions collective self-efficacy work. There can be such disparities and diverse constituencies that comprise even one neighborhood, let alone how a community is operationalized. Therefore, it would be beneficial to tease that out more. If there is some collective self-efficacy, what inside a community interacted or what were the prominent mechanisms of change under that bundle called “neighborhood” or “community?” This is not really a weakness per se but unpacking some of that may help to strengthen the study findings. With so many things going on around the proposed study, including the SAHMSA-funded parent program, it is not clear how the team will keep track of what this program and grant are doing in conjunction with or distinct from some of the other existing factors in the environment. There are some concerns about how the incidence of violence will be measured, given the heavy reliance on publicly available reports of community violence, like police and arrest records. Those reports alone can be unreliable. However, violence will be measured in two ways (i.e., through police offense arrest reports and through 911 calls), which strengthens the measure of violence.

Protection of human subjects is acceptable. Inclusion is acceptable in terms of gender, minority groups, and children. Animal welfare, biohazards, and dual use research are not applicable. The panel had varying views on the acceptability of the resource sharing/data management plan (DMP). Overall, this is an excellent application that is very strong with only some minor weaknesses.

DESCRIPTION (provided by applicant): Violence impacts the life trajectories of far too many adolescents, with the highest incidence of violence exposure concentrated among youth in urban settings. In response to RFA-CE-22-013, Rigorous Evaluation of Community-Centered Approaches for the Prevention of Community Violence (U01), this study will evaluate, via a cluster- randomized controlled trial, the effectiveness of a novel community-centered intervention that promotes thriving and resiliency to reduce community violence (CATEGORY 2). Using a community-partnered approach, we

will implement a Community Resiliency Collective Efficacy Intervention (CRCEI) to engage community members in dialogue on thriving, community leadership, and organizing for social change. Facilitating discussion and consensus organizing within neighborhoods about child and youth thriving is expected to increase individual and neighborhood levels of collective efficacy and reduce community violence. The proposal emerges from a SAM- HSA-funded community resiliency project in Pittsburgh (H79SM084931) that supports implementation of this novel community-centered approach. This study will provide the first rigorous evaluation of this innovative prevention approach. The project is set in urban, racially-segregated neighborhoods with concentrated disadvantage in Pittsburgh, Pennsylvania. We hypothesize that opportunities to co-create thriving environments for children and youth will provide a concrete, action-focused strategy for increasing resiliency in neighborhoods most impacted by community violence and reducing community rates of violence. The proposed project will engage diverse community members and collaborators in urban neighborhoods with high levels of community violence to first participate in six guided discussions about ‘child thriving’ followed by an invitation to participate in a 6-month-long collective efficacy intervention that promotes neighborhood-level transformation and a com- munity-based project. Comparison neighborhoods will receive health education sessions as a control intervention. A cluster-randomized controlled design (20 neighborhoods; 30 participants/neighborhood) will be used to assess effectiveness of CRCEI on neighborhood-level collective efficacy and rates of community violence (Aim 1), and individual-level collective efficacy and violence exposure (Aim 2). Implementation facilitators and barriers across individual and neighborhood level will be assessed, including tracking fidelity to intervention and costs associated with program implementation (Aim 3). Innovations of this project include implementing in trustworthy settings such as churches and libraries identified by community members using asset mapping, training facilitators from focus neighborhoods to build capacity and sustainability, and integrating community members into collaborative team science. This will be the first rigorous evaluation of a community-designed, community-centered collective efficacy intervention intended to impact individual and neighborhood levels of collective efficacy, protective factors that are expected to reduce multiple forms of violence.

PUBLIC HEALTH RELEVANCE: This study will examine, via a cluster-randomized controlled trial in 20 neighborhoods with concentrated disadvantage in Pittsburgh, Pennsylvania, the effectiveness of a community resiliency collective efficacy intervention. Using a community-partnered participatory approach, this study will assess an innovative approach to address community violence by engaging community members in dialogue on child and youth thriving and consensus organizing to build collective efficacy. This will be the first rigorous evaluation of a community-designed, community-centered collective efficacy intervention intended to increase individual and neighborhood levels of collective efficacy, protective factors that are expected to reduce multiple forms of violence.

CRITIQUE 1

Significance:	7
Investigator(s):	4
Innovation:	5
Approach:	8
Environment:	1

Overall Impact
Strengths

- The proposed study seeks funding to carry out a cluster RCT evaluation of a collective-efficacy intervention that is being implemented as part of a county-wide community resilience-building strategy to address community violence prevention. The key variable – collective efficacy – which is supposed to be built through community conversations and is expected to reduce rates of youth violence, has been identified as important for violence prevention in one of the Co-PIs previous studies. However, its relation to violence prevention has yet to be fully explored and

does not yet seem supported by previous research. The study is strong methodologically and could be valuable in potentially contributing another variable in understanding youth violence prevention.

Weaknesses

- The study is weak theoretically. There are many available models studying the causes of youth violence that include poverty, child maltreatment, substance abuse, untreated trauma, and unremediated learning disabilities (Zagar, Garbarino, etc.). Without those variables also being targeted by the intervention or at least measured, the relative strength of the community efficacy variable in predicting violence will not be ascertainable. The intervention seems likely to have weak impact in relation to those known triggers for violence. Because the sample size is large, the study may result in suggestive statistically significant relationships, but the lack of qualitative and theoretical data to understand the meaning of those connections will compromise the scientific contribution of the findings.
- The conceptual model and hypotheses should have some theoretical and empirical bases for focusing on the community efficacy variable in a predictive model including known causes of youth violence.
- The dosage and protective interventions are inadequate to the task of preventing violence engagement based on other studies about effective violence prevention.

1. Significance:

Strengths

- The project clearly addresses an extremely important problem, and the focus on strengthening communities is inherently valuable.

Weaknesses

- The target variable – community self-efficacy – while important, seems distant from the core triggers of violence which are known to be poverty, lack of educational preparedness for gainful employment such as can occur with unremediated learning disabilities, and child maltreatment.
- There is a concern that the key variable to be impacted by the intervention – collective self-efficacy – seems to assume that community members regard their difficulties as residing in inadequate collective self-efficacy. Movements such as BLM and the Third Reconstruction emphasize that problems in high-poverty, high-crime communities reside in wider social structures that perpetuate segregation, poverty, poor education, and mass incarceration. There is the question then whether community members would regard collective self-efficacy as more influential than those structural issues. There should be some evidence provided from community surveys that community members regard collective self-efficacy as valuable for violence prevention.
- Measures should also consider youth employment and high school/college graduation rates, especially among youth. Since remediating poverty is key to remediating violence, employment and educational resources are strong remedial variables but are not considered in their analyses.
- Planning to ensure engagement and reduce attrition in the high-poverty communities may be insufficient. Will the community members believe that the proposed intervention will solve problems of violence? The focus of TPS on compensating participants should help in that regard, as will the strong participatory elements, but engagement can be a major issue in communities where persons are struggling to meet basic survival needs, and those are the families whose members can be most prone to violence. How do we know those families most prone to violence will be included?

2. Investigator(s):

Strengths

- This is a very highly qualified team nested within several institutional supports that appear to be excellent both academically and in terms of infrastructure of research resources. The team includes a biostatistician with experience with cluster-based RCTs focused on adolescent violence and a clinician working to support youth post injury from violence. As such, the team appears to have in-depth knowledge of the problem area (Empowering Teens to Thrive).
- There is a strong network of community partnerships and a youth advisory board to support community engagement and relevance of the project.
- The PI is highly accomplished in scholarship, grant management, engaging community members, and ensuring community relevance of findings.
- More than the PIs, the Co-PIs (Ettinger, Fabio, Ohmer and Jacobs) have scholarship more in line with the Community Thriving focus of the proposal. Except for Ohmer, these are early career researchers whose work it is beneficial to support.

Weaknesses

- The PIs own scholarship seems more focused on reproductive coercion and gendered patterns of intimate partner violence. Clearly, she will lean heavily on the team from social work (Jacobs and Ohmer) to design and carry out interventions.
- There is concern about the PI being so stretched given several projects running now and for years into the future. It appears the funding is sought exclusively for the evaluation component, and primarily the research team, as it appears the services components are available through other sources, but this is not entirely clear. In other words, do the requested funds overlap with evaluation funds already available in concert with funding of services?

3. Innovation:

Strengths

- It is important to understand community-level variables that impact levels of violence. For instance, Co-PI Fabio's research about racial segregation contributing more than individual risk factors to community violence suggests this study could be innovative.

Weaknesses

- The community intervention model seems somewhat weak in relation to the depth of services known to be needed to prevent violence engagement (see for example intensive violence interruption program such as CureViolence and Safe Streets). The intervention may indeed yield community self-efficacy but that may not mean it prevents youth violence in any meaningful way. It is not clear how this conceptual model will contribute to existing models of young violence because while youth mental health is being measured, other known variables are not being measured (e.g., learning disabilities, substance abuse, and at the community level, trauma treatment mental health resources in communities).

4. Approach:

Strengths

- The RCT design is strong in terms of site selection and controls.
- The strong involvement of community members and the Youth Research Advisory Board, with compensation, speaks to understanding of community members' struggles and needs for support as well as relevance of research to their values and priorities.

Weaknesses

- Community intervention dosage is relatively weak (12 sessions) compared to dosages of other interventions known to reduce violence (e.g., trauma treatment models, youth primary prevention projects, etc.).

- Without specific remedies for poverty such as paths to education and employment included in the intervention, it does not have empirical warrant for promise in preventing violence.
- Without including families and youth specifically at risk for violence engagement or differentiating them for some specific intervention separate from those available for all community families, it is difficult to have meaningful, culturally relevant data about the connection between collective self-efficacy and preventing community youth violence. It is not sufficient to assume that residence in specific neighborhoods is equivalent to violence engagement, since studies by Papachristos, Anderson, and Kozol indicate the majority of community members in high-crime, high-poverty communities are not violence engaged. This is a key weakness in the study.
- Measures could be stronger. They should include more known triggers of youth violence as described above.
- Measuring the key dependent variable of incidence of violence is not easy. The project relies on publicly available reports of community violence as well as community members' reported experiences of safety in their neighborhoods. This is good triangulation since community members know of more violence than may be publicly available. However, public reports of violent crime are known to be unreliable. Likewise, community members can be attacked and not report the criminal act out of fear of retaliation by gang members and/or police. This problem should at least be addressed in the proposal since it poses a serious threat to validity.

5. Environment:
Strengths
- The environment is exceptionally strong.

Weaknesses
- No weaknesses are noted.

Protections for Human Subjects:
Acceptable. Human Subjects involved, no concern

Data and Safety Monitoring Plan (Applicable for Clinical Trials Only):
Not Applicable

Inclusion of Women, Minorites, and Age (Applicable Only for Human Subjects research and not IRB Exemption #4.):
G1A- Includes both genders, acceptable
M1A- Includes minorities & non-minorities, acceptable
C1A- Children, adults & older adults, acceptable

Vertebrate Animals:
Not Applicable

Biohazards:
Not Applicable

Dual Use Research of Concern:
Not Applicable

Budget and Period of Support:
Recommend as Requested

Resource Sharing Plans:
Acceptable

Data Management Plan (DMP):
Acceptable

CRITIQUE 2

Significance:	2
Investigator(s):	1
Innovation:	2
Approach:	1
Environment:	1

Overall Impact
Strengths
- The approach utilizes a randomized controlled trial.
- The study addresses racism and structural discrimination related to violence, which is important.
- This research really seems to involve the community.
- Focusing on community assets and strengths is worthwhile. Asset mapping is used to determine safe settings. Community members are facilitators.
- The analysis plan is very thorough and well thought out. Using multilevel modeling is a real strength in being able to look at both individual and neighborhood effects.

Weaknesses
- No weaknesses are noted.

1. Significance:
Strengths
- This study falls under Category 2 of the NOFO.
- This study proposes to evaluate an important strengths-based approach to oppressed communities.
- This proposal addresses racism through collective efficacy.

Weaknesses
- No weaknesses are noted.

2. Investigator(s):
Strengths
- Dr. Miller is a pediatrician and medical anthropologist with expertise in violence prevention. She has many funded projects in violence prevention. Dr. Miller has published an enormous amount in violence prevention.
- Dr. Abebe has a wealth of experience in violence prevention as a statistician. Dr. Abebe also has expertise in clinical trials (especially clustered RCTs).
- Dr. Culyba is a pediatrician and epidemiologist who has expertise in teen violence. She has experience with violence prevention interventions.
- All collaborators on this grant have excellent violence prevention expertise.
- There are a large number of community partners.

Weaknesses
- No weaknesses are noted.

3. Innovation:
Strengths

- The medical center already has a Youth Research Advisory Board which meets monthly to provide input on clinical and community-based research activities relevant to adolescent and young adult health.
- The intervention will be implemented in trustworthy settings (e.g., churches, libraries) identified by community members using asset mapping. Including trained facilitators from focus neighborhoods will help to build capacity and sustainability and integrate community members into the research process.
- The proposed study looks at both individual and neighborhood effects of collective efficacy on violence prevention.
- Addressing racism and discrimination is vitally important.
- This proposal aligns with CDC priorities.

Weaknesses

- No weaknesses are noted.

4. Approach:
Strengths

- The intervention takes place in segregated neighborhoods plagued by concentrated disadvantage.
- There are many community partners engaged in this work.
- The focus on racism and structural racism in the background and approach is important.
- Including youth on the steering committee and developing youth leadership skills is really valuable.
- Randomization is a strong study design.
- Violence is measured in two different ways (i.e., police offense/arrest reports and 911 calls) which strengthens the measure of violence, which is key here.
- The analysis plan is very thorough and well thought out. Using multilevel modeling is a real strength in being able to look at both individual and neighborhood effects.

Weaknesses

- No weaknesses are noted.

5. Environment:
Strengths

- The University of Pittsburgh seems to have ample space, facilities, and support to conduct much of this research including the PIs/Co-Is co-location, the CTSI, the Community Engagement and Research Core, the Youth Research Advisory Board, and many other centers.

Weaknesses

- No weaknesses are noted.

Protections for Human Subjects:
Acceptable. Human Subjects involved, no concern

Data and Safety Monitoring Plan (Applicable for Clinical Trials Only):
Not Applicable

Inclusion of Women, Minorites, and Age (Applicable Only for Human Subjects research and not IRB Exemption #4.):
G1A- Includes both genders, acceptable
M1A- Includes minorities & non-minorities, acceptable
C1A- Children, adults & older adults, acceptable

Vertebrate Animals:
Not Applicable

Biohazards:
Not Applicable

Dual Use Research of Concern:
Not Applicable

Budget and Period of Support:
Recommend as Requested

Resource Sharing Plans:
Unacceptable

Data Management Plan (DMP):
Unacceptable

CRITIQUE 3

Significance:	2
Investigator(s):	1
Innovation:	2
Approach:	2
Environment:	1

Overall Impact
Strengths
- Rigorously examining relationships between individual and collective efficacy both within and across different communities may offer important neighborhood-level data on mobilization and effort toward community-based violence prevention.
- This proposal makes efforts to mobilize multi-sector collaboration (including consumers/residents themselves) in neighborhood-level violence prevention.

Weaknesses
- The proposal needs further clarification of the research questions, methods, and possible outcomes of examining interpersonal processes and interactive factors (e.g., “how, why, and under what conditions”) that may result in successful neighborhood violence prevention efforts.

1. Significance:
Strengths
- The project proposes conducting a rigorous randomized research trial to examine impacts of collective efficacy and neighborhood dynamics toward community-based violence prevention.
- The study approach taps existing neighborhood institutions (e.g., libraries, faith-based institutions, etc.) in building neighborhood-based violence prevention collaboration.

Weaknesses
- The empirical investigation of not only “who” but “how, why, and under what conditions” these networks specifically and successfully mobilize around the topics and tasks related to violence prevention may have been further clarified.

2. Investigator(s):
Strengths

- The project will be housed in an existing research site that appears to have capacity to conduct the proposed study.
- The project will be conducted within an ongoing project and existing site that is currently part of an ongoing services research study – which further supports feasibility of research design and program delivery.

Weaknesses
- No weaknesses are noted.

3. Innovation:
Strengths
- This proposed study uses rigorous randomization with neighborhood-level variable considerations in examining community-based violence prevention.

Weaknesses
- Possible clarification of potential external impacts (e.g., influences from SAMHSA-funded parent program and other ongoing initiatives) on potential outcomes is not included.

4. Approach:
Strengths
- This study uses rigorous randomization in examining collective efficacy, social cohesion and connection, and neighborhood mobilization in risk and resiliency related to violence prevention.
- This study uses a balanced combination of existing and innovative data collection tools and methods (including mixed methodology).

Weaknesses
- The aggregate data analysis may obscure important individual variations within similarly assigned neighborhoods that offer additional evaluation insights.

5. Environment:
Strengths
- The proposed project leadership and location have a track record for conducting federally funded research trials (including CDC and NIH) along with foundation-funded studies on targeted topics.
- The proposed project leadership team has significant biostatistical, public health, and community-based professional experiences increasing the plausible implementation of this planned study.

Weaknesses
- No weaknesses are noted.

Protections for Human Subjects
Acceptable. Human Subjects involved, no concern

Data and Safety Monitoring Plan (Applicable for Clinical Trials Only):
Not Applicable

Inclusion of Women, Minorites, and Age (Applicable Only for Human Subjects research and not IRB Exemption #4.):
G1A- Includes both genders, acceptable
M1A- Includes minorities & non-minorities, acceptable
C1A- Children, adults & older adults, acceptable

Vertebrate Animals:

Not Applicable

Biohazards:
Not Applicable

Dual Use Research of Concern:
Not Applicable

Budget and Period of Support:
Recommend as Requested

Resource Sharing Plans:
Acceptable

Data Management Plan (DMP):
Acceptable

THE FOLLOWING SECTIONS WERE PREPARED BY THE SCIENTIFIC REVIEW OFFICER TO SUMMARIZE THE OUTCOME OF DISCUSSIONS OF THE REVIEW COMMITTEE, OR REVIEWERS' WRITTEN CRITIQUES, ON THE FOLLOWING ISSUES:

PROTECTION OF HUMAN SUBJECTS: ACCEPTABLE

INCLUSION OF WOMEN PLAN: ACCEPTABLE
Includes both genders, which is scientifically acceptable.

INCLUSION OF MINORITIES PLAN: ACCEPTABLE
Includes minorities and non-minorities, which is scientifically acceptable.

INCLUSION ACROSS THE LIFESPAN: ACCEPTABLE
Includes children, adults, and older adults, which is scientifically acceptable.

COMMITTEE BUDGET RECOMMENDATIONS: The budget is recommended as requested.
Note: This team has several projects running currently and for years into the future. It is possible that there might be overlap between this funding request and other projects being carried out by the PI and other investigators.

COMMITTEE RESOURCE SHARING PLAN RECOMMENDATIONS:
The panel had varying views on the acceptability of the Resource Sharing Plan.

COMMITTEE DATA MANAGEMENT PLAN RECOMMENDATIONS:
The panel had varying views on the acceptability of the Data Management Plan.

MEETING ROSTER

National Center for Injury Prevention Special Emphasis Panel
NATIONAL CENTER FOR INJURY PREVENTION AND CONTROL
CE22-013: Rigorous Evaluation of Community-Centered Approaches for the Prevention of Community Violence
ZCE1 MLW (13)
06/28/2022

CHAIRPERSON(S)
RENNER, LYNETTE MICHELLE, PHD
PROFESSOR
UNIVERSITY OF MINNESOTA
SCHOOL OF SOCIAL WORK
ST. PAUL, MN 55108

MEMBERS
ABDUL-ADIL, JALEEL S, PHD
ASSOCIATE PROFESSOR
UNIVERSITY OF ILLINOIS, CHICAGO
DEPARTMENT OF PSYCHIATRY
CHICAGO, IL 60612

ARMSTRONG, GAYLENE S, PHD
DIRECTOR AND PROFESSOR
UNIVERSITY OF NEBRASKA, OMAHA
SCHOOL OF CRIMINOLOGY AND CRIMINAL JUSTICE
OMAHA, NE 68182

ATLAS, RANDY, PHD
PRESIDENT
ATLAS SAFETY & SECURITY DESIGN INC.
FORT LAUDERDALE, FL 33301

FRANCOIS, SAMANTHA, PHD
ASSISTANT PROFESSOR
TULANE UNIVERSITY
NEW ORLEANS, LA 70112

GRINSHTEYN, ERIN, MS, BA, PHD
ASSOCIATE PROFESSOR
UNIVERSITY OF SAN FRANCISCO
SCHOOL OF NURSING AND HEALTH PROFESSIONS
SAN FRANCISCO, CA 94177

MCCREA, KATHERINE TYSON, MDIV, HONOR PHD, MSW, PHD
PROFESSOR
LOYOLA UNIVERSITY CHICAGO
SCHOOL OF SOCIAL WORK
CHICAGO, IL 60611

SCIENTIFIC REVIEW OFFICER
WALTERS, MIKEL, MA, PHD
ACTING DIRECTOR, EXTRAMURAL RESEARCH PROGRAM OPERATIONS
NATIONAL CENTER FOR INJURY PREVENTION & CONTROL
NATIONAL CENTER FOR ENVIRONMENTAL HEALTH/ATSDR
CENTERS FOR DISEASE CONTROL AND PREVENTION
ATLANTA, GA 30341

EXTRAMURAL SUPPORT ASSISTANT
ROBERTS, KENNETH H
GRANTS TECHNICAL ASSISTANT
NATIONAL CENTER FOR INJURY PREVENTION & CONTROL
NATIONAL CENTER FOR ENVIRONMENTAL HEALTH/ATSDR
EXTRAMURAL RESEARCH PROGRAM OFFICE
CENTERS FOR DISEASE CONTROL & PREVENTION
ATLANTA, GA 30341

Consultants are required to absent themselves from the room during the review of any application if their presence would constitute or appear to constitute a conflict of interest.
